# The Effects of a SWELE Program on Mental Wellbeing Among Children and Adolescents With Special Educational Needs and Disabilities: A Quasi‐Experimental Study

**DOI:** 10.1111/jar.70164

**Published:** 2025-12-18

**Authors:** Regina Lai Tong Lee, Alice Wai Yi Leung, Yuen Yu Chong, Sally Wai Chi Chan, Kai Chow Choi, Wai Tong Chien

**Affiliations:** ^1^ The Nethersole School of Nursing, Faculty of Medicine The Chinese University of Hong Kong Hong Kong; ^2^ School of Nursing and Midwifery, College of Health, Medicine & Wellbeing University of Newcastle Newcastle New South Wales Australia; ^3^ Tung Wah College Hong Kong

**Keywords:** anxiety, children and adolescents, mindfulness interventions, mood, playfulness, self‐regulation, social skill, special educational needs, unstructured play

## Abstract

**Background:**

The aim was to examine the effects of ‘Supporting Wellness in E‐Child Learning Environments’ (SWELE) program on the mood, anxiety, social and self‐regulation skills and playfulness behaviours of children and adolescents with special educational needs and disabilities.

**Methods:**

A quasi‐experiment single‐group pre‐test and post‐test design was adopted. The intervention group received a 16‐week SWELE program featuring an unstructured free outdoor play coupled with mindfulness‐based intervention.

**Results:**

1187 children and adolescents with intellectual or physical disabilities were recruited. At post‐intervention, there are consistent positive changes across all five outcomes with medium to large effect sizes (Cohen's d: 0.63–0.80). Specifically, the SWELE program demonstrated statistically significant improvement in mood level (*t* = 21.86, *p* < 0.001), self‐regulation skills (*t* = 29.59, *p* < 0.001) and social skills (*t* = 23.84, *p* < 0.001), reduction in anxiety symptoms (*t* = −27.47, *p* < 0.001), and an increase in playfulness behaviours (*t* = 20.84, *p* < 0.001).

**Conclusion:**

The SWELE intervention program was effective in promoting mental wellbeing among target children and adolescents.

## Lay Summary

- The SWELE mental health promotion program demonstrated statistically significant improvement in mood level, self‐regulation and social skills, anxiety symptoms, and playfulness behaviours among SEND children and adolescents.
- The SWELE program's play‐based approach, combining unstructured outdoor activities with mindfulness‐based interventions, appears to be a beneficial strategy for promoting mental wellbeing and emotional competency in this population with special educational needs.
- The study findings provide new evidence for policy makers and health planners to develop relevant mental health promotion programs and services for SEND children and adolescents.

## 1 Introduction

Children with special educational needs and disabilities (SEND) tend to have lower levels of emotional literacy and limited opportunities to deal with their mood disturbance, anxiety and antisocial behaviours, and coping skills (Unwin et al. 2018). SEND encompass a wide range of conditions including intellectual and physical disabilities. Intellectual disability is defined as a developmental disability with learning needs arising from various types of physical and mental illnesses or conditions with significant limitations in both intellectual functioning and adaptive behaviour as expressed in conceptual, social, and practical skills (Schalock et al. 2021). The common types of physical disability include spina bifida, spinal muscular atrophy, cerebral palsy, juvenile rheumatoid, osteogenesis imperfecta, muscular dystrophy and cerebral palsy (Education Bureau 2021b). Children and adolescents with SEND are at increased risk of experiencing varying degrees of mental health challenges before 22 years old. These challenges may manifest as mood disorders, anxiety, distress, psychological discomfort, difficulty with self‐regulation, poor social relationship with peers, significant delays in reaching developmental milestones (Blanc et al. 2005; McMillan and Jarvis 2013; McNamara et al. 2018). However, there remains a lack of targeted interventions that effectively promote the mental wellbeing among this vulnerable population although previous studies have shown that emotional and social learning interventions are critical to support the development of intra‐ and inter‐personal skills to promote socio‐emotional wellbeing for all students in the school settings (Cipriano et al. 2023). The emotional and social aspects of each individual constitute of mental state and behaviours as they encounter life challenges, make decisions and build relationships which affect all aspects of a person's life (Lee 2011; National Research Council Institute of Medicine 2009).

Play supports healthy brain development and allows children and adolescents to explore their fears and build relationships with their peers and adults. Jean Piaget's Theory of Play asserted that children's cognitive development and play are keenly intertwined as children learn better when they explore new things around them and make use of all their senses during play (Piaget 1951). Play is known as the core occupation among young children as it lays a foundation for life skills for their early childhood development in at least one of these domains: physical health, social competence, mental wellbeing and emotional stability (Bundy et al. 2011; Lee, Lane, Brown, et al. 2020; Lee, Lane, Tang, et al. 2020; Nijhof et al. 2018).

The benefits of play in reducing anxiety and enhancing emotional wellbeing among children with chronic medical illness have been informed (He et al. 2015; LaFreniere 2013). Unstructured play activities have been found to be effective in promoting positive emotions and emotional competence among SEND children and adolescents (Francis et al. 2022). On the other hand, recent literature reviews reported positive effects of mindfulness‐based programs on the development of self‐regulation skills in young children (Bockmann and Yu 2023; Sop and Hançer 2024). A recent randomized control trial showed that an embodied mindfulness‐based movement programme enhanced attention in children with cerebral palsy compared with a waitlist comparison group (Mak et al. 2018).

Despite the potential benefits of unstructured play and mindfulness‐based interventions on the mental well‐being of children, few studies have integrated unstructured play with mindfulness‐based interventions and none have examined the effects of such interventions on the mental wellbeing of children and adolescents with SEND (Bockmann and Yu 2023; Francis et al. 2022; Lee, Lane, Brown, et al. 2020; Lee, Lane, Tang, et al. 2020; Mak et al. 2018; Sop and Hançer 2024). To fill this important gap, our research team has developed an unstructured outdoor play activities coupled with mindfulness‐based intervention via ‘Supporting Wellness in E‐Child Learning Environments’ (SWELE) program to foster the mental wellbeing among SEND children and adolescents (reference blinded for review). The conceptual framework of Bandura's social cognitive theory (Bandura 1977) was adopted in designing this behavioural change intervention via SWELE program coupled with mindfulness interventions (reference blinded for review). Thus, the aim of this study is to examine the effects of SWELE program on the SEND children's and adolescents' mental wellbeing including mood level, anxiety symptoms, social and self‐regulation skills, and playfulness behaviours as the primary outcomes of this study.

## 2 Methods

### 2.1 Design

This study employed a quasi‐experimental single‐group pre‐test and post‐test design. We implemented the SWELE program coupled with mindfulness interventions in seven government‐funded special schools in Hong Kong between September 2023 and July 2024. Pre‐test and post‐test assessments were completed in the seven special schools with pre‐test collecting baseline data before the SWELE program implementation and post‐test conducted immediately after program completion. The protocol of this study has been published elsewhere (reference blinded for review).

#### 2.1.1 Trial Registration

This study was registered in ClinicalTrial.gov on 31 October 2023 with trial registration number (NCT06112483). The reporting of this study adheres to the TREND Statement Checklist (Des Jarlais et al. 2004).

### 2.2 Participants

The Education Bureau of Hong Kong Government has categorized nine common types of SEND in children including intellectual disability, autism spectrum disorder, attention deficit hyperactivity disorder, mental illness, specific learning difficulties, physical disability, visual impairment, hearing impairment and speech and language impairment (Education Bureau 2021a). In this study, we recruited SEND children and adolescents with either mild or moderate intellectual disability or physical disability from seven government‐funded special schools to participate in the SWELE program. The participants were recruited in two batches between September 2023 and July 2024. The first batch included three special schools with intellectual disability, and the second batch included four special schools with intellectual disability and/or physical disability. Grade 1 to Grade 12 SEND students (children and adolescents) were enrolled from seven participating special schools in Hong Kong.

The inclusion criteria for study participants included SEND children and adolescents aged 6–19 years who (1) were enrolled in a special school; (2) could speak and understand Cantonese; (3) had no diagnosis of any cardiovascular disease; (4) had obtained parental consent. The exclusion criteria included those who (1) were not enrolled in a special school; (2) could not speak and understand Cantonese; (3) had a diagnosis of cardiovascular disease; (4) were not able to provide parental consent.

The sample size was anticipated to be at least 850 children, based on the assumption that at least 90% would be eligible and willing to join and the attrition rate would be under 10%. This sample size allows us to detect an effect size as small as 0.1 with 80% power at a significance level of 0.05 (2 sided) (Cohen 1992).

### 2.3 SWELE Program

The SWELE program was originally developed and piloted with children aged four to six years in Hong Kong in 2020. During the pilot study, we only conducted the intervention for five consecutive days due to limited resources and funding. The findings showed potential benefits in promoting children's happiness and playfulness (Lee, Lane, Tang, et al. 2020). In this study, we extended the program to 16 weeks and adapted it to promote mental wellbeing among SEND children and adolescents in the special schools. This program combined with mindfulness‐based intervention was grounded in concepts of freedom to learn and freedom to play by encouraging independence and exploration. Unstructured play allows children the freedom to explore, create and discover without predetermined rules or guidelines. It has been shown to foster cognitive development while boosting physical development and social and emotional development. It specifically helps creativity and imagination, problem‐solving abilities and social skills. Mindfulness training positively impacts cognitive performance for learning by enhancing attention, concentration, and working memory, while reducing mind wandering and stress, which collectively improve academic achievement and overall mental well‐being. Details of the SWELE program has been described in Table 1 and the protocol (reference blinded for review). There was no protocol deviations from the study as planned.

**TABLE 1:** A 16‐week SWELE program–extracurricular activities to promote SEND children's and adolescents' mental wellbeing status.

Week	SWELE activities for age‐appropriate groups/classes	Frequency/Schedule	Venue
Week 1 & 2 (2 days per week)	Practicing mindfulness activities (deep breathing technique, yoga, story ‐telling, mindful arts, eye‐closing with calmness music for relaxation)	30 min each session 3 h TOT Workshop	School hall
	Training Workshop for Youth Mental Health Ambassador Program (SEND Students)		School playground
Week 3 & 4 (2 days per week)	Practicing mindfulness activities (deep breathing technique, yoga, mindful arts, storytelling, eye‐closing with calmness music for relaxation)	10 min each session	School playground
	Outdoor playground age‐appropriate activities/games (natural and recycle materials: branches, tyres, sand, water, leaves, boxes, plastic bottles, tools/toys…)	45–60 min each session	
Week 5 & 6 (2 days per week)	Practicing mindfulness activities (deep breathing technique, yoga, story ‐telling, mindful arts, eye‐closing with calmness music for relaxation)	10 min each session	School playground
	Tunnel‐through‐boxes, Tyres, Sand and water play, Tree House & Adventure Course, Wobbly Wood Oasis Hut, Merchant Play Ship with Pirate Tower. Play Ship Nina, Sky climber with Artwork	45–60 min each session	
Week 7 & 8 (2 days per week)	Practicing mindfulness activities (deep breathing technique, yoga, mindful arts, eye‐closing with calmness music for relaxation)	10 min each session	School hall
	Outdoor playground age‐appropriate activities/games (natural and recycle materials: branches, tyres, sand, water, leaves, boxes…)	45–60 min each session	School playground
Week 9 & 10 (2 days per week)	Practicing mindfulness activities (deep breathing technique, yoga, mindful arts, eye‐closing with calmness music for relaxation)	10 min each session	School hall
	Mental Health Campaign (Games, smiling stickers, posters, emoji)	45–60 min each session	
Week 11 & 12 (2 days per week)	Practicing mindfulness activities (deep breathing technique, yoga, mindful arts, eye‐closing with calmness music for relaxation)	10 min each session	School hall
	Outdoor playground age‐appropriate activities/games (natural and recycle materials: branches, tyres, sand, water, leaves, boxes, plastic bottles…)	45–60 min each session	School playground
Week 13 & 14 (2 days per week)	Practicing mindfulness activities (deep breathing technique, yoga, mindful arts, storytelling, eye‐closing with calmness music for relaxation)	10 min each session	School hall
	Outdoor playground age‐appropriate activities/games (natural and recycle materials: branches, tyres, sand, water, leaves, boxes, plastic bottles, tools/toys.)	45–60 min each session	School playground
Week 15 & 16 (2 days per week)	Practicing mindfulness activities (deep breathing technique, yoga, mindful arts, storytelling, eye‐closing with calmness music for relaxation)	10 min each session	School hall
	Outdoor playground age‐appropriate activities/games (natural and recycle materials: branches, tyres, sand, water, leaves, boxes, plastic bottles, tools/toys…)	45–60 min each session	School playground

Briefly, the SWELE program team including researchers and research assistants planned age‐appropriate unstructured outdoor free play and mindfulness‐based activities according to the schedules provided by each school. All activities were conducted either in the school hall or playground (Table 1). The 10‐min mindfulness‐based activities included podcasts and art, along with meditation, deep breathing technique, storytelling accompanied by relaxing piano music and mindful art. The 30–40 min unstructured play included creative activities like sand, water, branches, boxes, artistic or musical games, imaginative play, and exploration of familiar spaces such as backyards of school playground. Additionally, training workshops were conducted for scouts enrolled as Youth Mental Health Ambassadors to equip them with knowledge and skills needed in assisting the research assistants to implement the intervention activities in each special school.

The intervention activities allowed SEND children and adolescents to explore, create and discover freely without predetermined rules or guidelines, as well as to facilitate social interactions and improvement of social skills. The SWELE program aimed to promote healthier environments, integrate mental health wellness practices into play activities, and support educators and social workers to promote mental health and emotional wellbeing for SEND children and adolescents in each special school two times per week. This comprehensive approach not only supports children's immediate health needs but also lays a foundation for addressing long‐term mental health challenges, potentially reducing issues such as anxiety, emotional regulation difficulties, and stress‐related disorders (reference blinded for review).

### 2.4 Outcome Measures

#### 2.4.1 Demographics

The demographic information included age, gender, grade, race and types of SEND schools were collected by trained research assistants. Mood, anxiety symptoms, self‐regulation and social skills, and playfulness behaviours were assessed through observations and filling of questionnaires by trained research assistants and the assistance with the schoolteachers.

#### 2.4.2 Emoji Mood Scale

To be inclusive and accurately capture each SEND student's self‐report on mood and emotion, it is useful to present visual stimuli in association with text. The Emoji Mood Scale includes five different emoji arranged on 5‐point scale for assessing mood levels: 1‐very bad, 2‐bad, 3‐so‐so, 4‐good and 5‐very good (Hall et al. 2016). A higher score represents better mood and more positive emotion.

#### 2.4.3 Six‐Item Short‐Form of State–Trait Anxiety Inventory (STAI)

The 6‐item short‐form version of the STAI is a validated measure sensitive to fluctuations in state anxiety (Marteau and Bekker 1992). When compared with the full‐form of the STAI, the six‐item version offers a briefer yet acceptable scale for subjects. This six‐item short‐form version produce scores similar to those obtained using the full‐form version. Observers were asked to rate each item using a Likert scale of 1–5: 1 = not at all/very slightly, 2 = a little, 3 = moderately, 4 = quite a bit, and 5 = extremely. The scores are summed to give a total score ranging from 6 to 24. A higher score shows a higher level of anxiety of individual. The Chinese version of the STAI‐6 demonstrated sound psychometric properties among Chinese children (Li and Lopez 2007).

#### 2.4.4 Child Behaviour Rating Scale (CBRS)

The Child Behaviour Rating Scale (CBRS) is a teacher‐report scale that assesses self‐regulation skills and social skills (Marteau and Bekker 1992). This scale comprises 17 items categorized into two subscales: the 10‐item classroom self‐regulation subscale assessing teachers' perceptions of children's behavioural regulation during academic tasks, and the 7‐item social skills subscale assessing teachers' perceptions of children's behavioural regulation in social situations. The Chinese version of CBRS has been validated by Chan et al. (2021).

#### 2.4.5 | Children's Playfulness Scale (CPS)

The Children's Playfulness Scale (CPS) is used to assess children's playfulness based on five dimensions of play among SEND students (Lieberman 1977). Lieberman proposed five manifestations of playfulness for the CPS: physical spontaneity, social spontaneity, cognitive spontaneity, manifest joy, and sense of humour (Lieberman 1977). Barnett developed a 23‐item questionnaire based on these five dimensions of play (Barnett 1990). Each item poses a statement such as “the child uses unconventional objects in play,” which is scored on a 5‐point Likert‐type scale with responses ranging from “sounds exactly like the child” to “doesn't sound at all like the child” (Barnett 1990). The raters respond to each statement by choosing one of five response alternatives. (Barnett 1990). The CPS instrument has been shown to be reliable and valid with inter‐rater reliability among the trained teachers exceeding 0.87 (Barnett 1990).

### 2.5 Statistical Analysis

Statistical analyses were performed using SPSS version 29 (IBM Corp., Armonk, NY). Data were summarized and presented using appropriate descriptive statistics. Normality of the continuous variables, including the outcome scores, was assessed based on their skewness and kurtosis statistics and normal probability plots. No continuous variables found violated normality. Paired t‐tests was used to compare pre‐test and post‐test scores for each outcome measure. Comparison of outcomes were made between the primary and secondary sections. Cohen's d for correlated measurements was also calculated to quantify the magnitude of effect on each outcome (Cohen 1992). All statistical test involved were two‐sided with level of significance set at 0.05.

### 2.6 Ethical Considerations

Ethical approval was obtained from the (name blinded for review) clinical research ethics committee prior to conducting the study (CREC ref. no: 2022.659‐T).

Parental consents were obtained from seven participating special schools prior to baseline data collection. The parents were given an information sheet and a consent form via each special school's communication system.

## 3 Results

### 3.1 Characteristics of Study Participants

Table 2 provide an overview of the characteristics of the study participants, with their age, gender, level of education (grades in the primary and secondary sections), race and types of disabilities (intellectual or physical). A total of 1208 participants were invited to participate the SWELE program, however, 21 participants could not provide the parental consents. Finally, a total of 1187 SEND children and adolescents aged 6–19 years old were recruited from the seven special schools in both primary section (*n* = 668) and secondary section (*n* = 519) in two batches between September 2023 and July 2024. Majority of the participants were recruited from special schools with intellectual disability (74.1%) and were males (73%). Mean age of the participants was 11.79 (SD = 3.45). Most of the participants were Chinese (97.2%). No missing data was found in this study as the schoolteachers assisted the data collection process. No adverse events were reported in this study.

**TABLE 2:** Overall demographic information of SEND children and adolescents participating in a 16‐week SWELE program by (a) primary and secondary sections and (b) by gender.

(a) Demographic variables	Primary section (*n* = 729)	Secondary section (*n* = 458)	All (*N* = 1187)
	Mean (SD)	Mean (SD)	Mean (SD)
Age, in years	9.27 (1.76)	15.03 (2.14)	11.79 (3.45)
Gender	*n* (%)	*n* (%)	*n* (%)
Male	503 (75.3)	364 (70.1)	867 (73.0)
Female	165 (24.7)	155 (29.9)	320 (27.0)
Ethnicity	*n* (%)	*n* (%)	*n* (%)
Chinese	711 (97.5)	443 (96.7)	1154 (97.2)
South Asian	10 (1.4)	9 (2.0)	19 (1.6)
Pakistani	8 (1.1)	6 (1.3)	14 (1.2)
Types of special schools (SEND)	*n* (%)	*n* (%)	*n* (%)
Intellectual Disability (ID)	514 (58.4)	366 (41.6)	880 (74.1)
Physical Disability (PD)	154 (50.2)	153 (49.8)	307 (25.9)

(b) Demographic variables	Males (*n* = 867)	Females (*n* = 320)	All (*N* = 1187)
	Mean (SD)	Mean (SD)	Mean (SD)
Age, in years	11.71 (3.44)	12.01 (3.49)	11.79 (3.45)
Age groups	*n* (%)	*n* (%)	*n* (%)
6–8	184 (21.2)	52 (16.3)	236 (19.9)
9–11	260 (30.0)	99 (30.9)	359 (30.2)
12–14	225 (26.0)	84 (26.3)	309 (26.0)
15–17	137 (15.8)	64 (20.0)	201 (16.9)
18–19	61 (7.0)	21 (6.6)	82 (6.9)
Grades	*n* (%)	*n* (%)	*n* (%)
Primary section			
Grade 1–3	203 (23.4)	67 (20.9)	270 (22.7)
Grade 4–6	300 (34.6)	98 (30.6)	398 (33.5)
Secondary section			
Grade 7–9	217 (25.0)	92 (28.7)	309 (26.0)
Grade 10–12	147 (17.0)	63 (19.7)	210 (17.7)

### 3.2 Pre‐Test and Post‐Test Comparison of the Mood

After the implementation of the SWELE program, there was a significant improvement in participants' mood level (Table 3). Post‐test mood scores (M = 4.23, SD = 0.76) were significantly higher than pre‐test scores (M = 3.54, SD = 0.92), with a mean increase of 0.69 points (*t* = 21.86, *p* < 0.001). This improvement was observed across both the primary section (mean difference = 0.73) and secondary section (mean difference = 0.63) in the seven special schools. The effect size, measured by Cohen's d for correlated measurements, was 0.63 for the overall sample, indicating a medium to large practical significance. The consistency of this positive change across sections and the statistical significance (*p* < 0.001) strongly support the effectiveness of the intervention in enhancing participants' mood levels.

**TABLE 3:** Comparison of mood level among SEND children and adolescents before and after participating in a 16‐week SWELE program.

	Primary section (*n* = 668)		Secondary section (*n* = 519)		All (*N* = 1187)	
	Mean (SD)	Range	Mean (SD)	Range	Mean (SD)	Range
Mood level						
Pre‐test	3.55 (0.90)	1–5	3.53 (0.96)	1–5	3.54 (0.92)	1–5
Post‐test	4.28 (0.72)	2–5	4.16 (0.81)	1–5	4.23 (0.76)	1–5
Mean difference	0.73		0.63		0.69	
*t*, *p* value	17.77***, < 0.001		12.99***, < 0.001		21.86***, < 0.001	
Cohen's *d*	0.69		0.57		0.63	

*Note:* ***p < 0.001. Emoji for mood scale consists of 1 item; scores ranged from 1 to 5 (1‐very bad; 2‐bad, 3‐so‐so, 4‐good; 5‐very good).

### 3.3 Pre‐Test and Post‐Test Comparison of the Anxiety Symptoms

After the implementation of SWELE program, there was a significant reduction in participants' anxiety levels (Table 4). Post‐test anxiety scores (M = 19.67, SD = 8.05) were significantly lower than pre‐test scores (M = 28.38, SD = 9.25), with a substantial mean decrease of 8.71 points (*t* = −27.47, *p* < 0.001). This reduction was consistent across both the primary section (mean difference = −8.74) and secondary section (mean difference = −8.96) in the seven special schools. The effect size, measured by Cohen's d, was 0.80 for the overall sample, indicating a large practical significance. The magnitude of this change, its consistency across sections, and the high statistical significance (*p* < 0.001) provide strong evidence for the effectiveness of the intervention in reducing anxiety levels among participants.

**TABLE 4:** Comparison of anxiety level among SEND children and adolescents before and after participating in a 16‐week SWELE program.

	Primary section (*n* = 668)		Secondary section (*n* = 519)		All (*N* = 1187)	
	Mean (SD)	Range	Mean (SD)	Range	Mean (SD)	Range
Anxiety level						
Pre‐test	27.65 (9.22)	13–52	29.32 (9.31)	13–52	28.38 (9.25)	13–52
Post‐test	18.91 (7.42)	13–51	20.66 (8.23)	13–52	19.67 (8.05)	13–52
Mean difference	−8.74		−8.66		−8.71	
*t*, *p* value	−21.10***, < 0.001		−17.62***, < 0.001		−27.47***, < 0.001	
Cohen's *d*	0.82		0.77		0.80	

*Note:* ***p < 0.001. State–Trait Anxiety Inventory consists of 13 items; score ranged from 13 to 52, the lower the scores, the lower level of anxiety.

### 3.4 Pre‐Test and Post‐Test Comparison of Self‐Regulation and Social Skills

The SWELE program demonstrated significant improvement in both the self‐regulation skills and social skills among participants (Table 5). For the self‐regulation skills, post‐test self‐regulation scores (M = 37.74, SD = 8.90) were significantly higher than pre‐test scores (M = 29.10, SD = 7.96), with a substantial mean increase of 8.65 points (*t* = 29.59, *p* < 0.001). This improvement was consistent across both primary section (mean difference = 9.00) and secondary section (mean difference = 8.20) in the seven special schools. The effect size, measured by Cohen's d, was 0.86 for the overall sample, indicating a large practical significance.

**TABLE 5:** Comparison of self‐regulation and social skills among SEND children and adolescents before and after participating in a 16‐week the SWELE program.

	Primary section (*n* = 668)		Secondary section (*n* = 519)		All (*N* = 1187)	
	Mean (SD)	Range	Mean (SD)	Range	Mean (SD)	Range
Self‐regulation skills						
Pre‐test	29.07 (7.51)	10–50	29.13 (8.51)	10–50	29.10 (7.96)	10–50
Post‐test	38.06 (7.51)	10–50	37.33 (9.26)	10–50	37.74 (8.90)	10–50
Mean difference	9.00		8.20		8.65	
*t*, *p* value	23.26***, < 0.001		18.40***, < 0.001		29.59***, < 0.001	
Cohen's *d*	0.90		0.81		0.86	
Social skills						
Pre‐test	18.69 (4.88)	7–34	18.49 (5.27)	7–35	18.60 (5.05)	7–35
Post‐test	23.10 (4.89)	7–35	22.44 (4.99)	7–35	22.81 (4.94)	7–35
Mean difference	4.41		3.96		4.21	
*t*, *p* value	18.72***, < 0.001		14.82***, < 0.001		23.84***, < 0.001	
Cohen's *d*	0.72		0.65		0.69	

*Note:* ***p < 0.001. Child Behaviour Rating Scale (CBRS)–the subscale of self‐regulation consists of 10 items, score ranged from 10 to 50, the higher the scores, the higher level of self‐regulation skills; and subscale of social skills consists of 7 items, score ranged from 7 to 35, the higher the scores, the higher level of social skills.

For the social skills, the post‐test social skills scores (M = 22.81, SD = 4.94) were also significantly higher than pre‐test scores (M = 18.60, SD = 5.05), with a mean increase of 4.21 points (*t* = 23.84, *p* < 0.001). This improvement was also consistent across the primary section (mean difference = 4.41) and secondary section (mean difference = 3.96) in the seven special schools. The effect size (Cohen's d = 0.69) indicates a medium to large practical significance.

### 3.5 Pre‐Test and Post‐Test Comparison of the Playfulness Behaviours

The SWELE program resulted in a significant enhancement of participants' playfulness behaviours as shown in Table 6. Post‐test playfulness behavioural scores (M = 76.28, SD = 18.16) were significantly higher than pre‐test scores (M = 63.96, SD = 15.92), with a substantial mean increase of 12.32 points (*t* = 20.84, *p* < 0.001). This improvement was consistent across both primary section (mean difference = 12.81) and secondary section (mean difference = 11.68) in the seven special schools. The effect size, measured by Cohen's d, was 0.61 for the overall sample, indicating a medium to large practical significance.

**TABLE 6:** Comparison of playfulness behaviours among SEND children and adolescents before and after participating in a 16‐week SWELE program.

	Primary section (*n* = 668)		Secondary section (*n* = 519)		All (*N* = 1187)	
	Mean (SD)	Range	Mean (SD)	Range	Mean (SD)	Range
Playfulness behaviours						
Pre‐test	64.79 (15.33)	26–108	62.90 (16.60)	24–111	63.96 (15.92)	24–111
Post‐test	77.60 (17.08)	30–113	74.58 (19.35)	28–115	76.28 (18.16)	28–115
Mean difference	12.81		11.68		12.32	
*t*, *p* value	16.33***, < 0.001		13.00***, < 0.001		20.84***, < 0.001	
Cohen's *d*	0.63		0.57		0.61	

*Note:* ***p < 0.001. Children's Playfulness Scale (CPS) consists of 23 items, score ranged from 23 to 115, the higher the scores, the higher level of playfulness behaviours.

The substantial increase in mean scores demonstrates that participants exhibited notably higher levels of playfulness after the SWELE program. The consistency of this positive change across sections, coupled with the high statistical significance (*p* < 0.001) and meaningful effect size, provides preliminary evidence for the effectiveness of the intervention in enhancing participants' playfulness behaviours. This improvement in playfulness behaviours could have important implications for children's overall development of health and well‐being of children and adolescents, as playfulness behaviours are often associated with creativity, social skills, and emotional regulation.

## 4 Discussion

Overall, the study findings supported the positive effects of the SWELE program via unstructured free play activities combined with mindfulness‐based intervention on all the five outcome measures of mental wellbeing among the children and adolescents with SEND in the seven participating special schools. Remarkably, there were consistent positive changes across all measured outcomes in both primary and secondary sections, along with medium to large effect sizes. The positive findings are similar to those studies that implemented school‐based mental health promotion programs to improve emotional, social and coping skills among children and adolescents in special schools (Unwin et al. 2018) and ordinary schools in European and other countries (Cefai et al. 2022; Colomeischi et al. 2022).

For mood enhancement, the post‐test mood scores were significantly higher than pre‐intervention scores, with a medium to large practical significance. This improvement in mood was consistent across both primary and secondary sections, suggesting that the SWELE program's effects in enhancing emotional wellbeing across age groups in both primary and secondary sections among the SEND children and adolescents. This finding aligns with the emerging evidence that supports the effects of play‐based or mindfulness‐based interventions on enhancing emotional regulation or mood among children (Francis et al. 2022; Mak et al. 2019).

Regarding anxiety symptoms, the SWELE program in this study led to a substantial decrease in anxiety levels. Post‐test anxiety scores were significantly lower than pre‐test scores, with a large effect size underscoring the program's notable impact on reducing anxiety symptoms among SEND children and adolescents. Similar findings have been noted in mindfulness‐based or relaxation interventions among individuals with a learning disability (Beaudoin et al. 2024).

For self‐regulation and social skills, there were significant improvements in both skills with medium to large effect sizes. These results are supported by a meta‐analysis of school‐based social–emotional learning interventions across 53 countries which aimed to develop advanced social skills to foster meaningful relationships with their peers and adults (Cipriano et al. 2023). Additionally, a review of 17 studies on the effectiveness of mindfulness interventions in self‐regulation skills among children found that mindfulness can strengthen children's brain regions for self‐control in emotional regulation, thereby reducing negative emotions and behaviours (Sop and Hançer 2024).

For playfulness behaviours, the SWELE program also led to significant increases in playfulness behaviours with a medium to large effect size. This improvement in playfulness behaviours could have important implications for SEND children's and adolescents' overall developmental milestones. This finding echoed those reported in the Sydney playground project and SWELE kindergarten study, which demonstrated that unstructured play in addition to mindfulness intervention effectively enhanced children's playfulness level (Bundy et al. 2011) (another reference blinded for review).

The mental wellbeing of SEND children and adolescents continues to be a global concern. There has been growing evidence on the effects of play‐based or mindfulness‐based interventions on mental wellbeing of children (Bockmann and Yu 2023; Francis et al. 2022; Lee, Lane, Brown, et al. 2020; Lee, Lane, Tang, et al. 2020; Mak et al. 2018; Sop and Hançer 2024); however, studies focusing on SEND children are largely lacking. Unstructured outdoor play can facilitate SEND children and adolescents to recognise and express their emotions and develop positive relationships with peers and family members. It also helps them to deal with anxiety and boredom and build their ability to concentrate and focus on what's important to them (Lee, Lane, Brown, et al. 2020). Mindfulness draws awareness of individuals' thoughts and actions in the present moment. It is recognized for helping young children improve attention, regulate behaviours, and social relationships by enhancing their awareness of feelings and thoughts (Sop and Hançer 2024). The success of SWELE program is likely the synergetic effect of unstructured free play coupled with mindfulness interventions in promoting mental health and wellbeing for this vulnerable group. The five outcomes represent various aspects of mental wellbeing of the SEND children and adolescents. As such, our findings add evidence to the effects of combining unstructured free play and mindfulness training on psychological health among SEND children and adolescents. Further research is warranted to confirm the effectiveness and long‐term effects of the SWELE program, as well as its potential for broader implementation in various settings and among different population groups.

Many stakeholders do not fully understand the concept of unstructured free play coupled with mindfulness interventions and the benefits it offers to young children, especially in promoting children's mental wellbeing. A UNICEF study reported that about 84% of parents misunderstood what free play meant. Children and adolescents need the space to set the rules and direction of their learning for the activity to count as free play (Chiu 2018). School health policy should ensure that the school‐based integrated curriculums provide an ideal environment for social and emotional learning to take place in assisting students to build essential life skills (Clarke et al. 2015). With the positive effects demonstrated in this program, it is anticipated that the SWELE program featuring unstructured free play coupled with mindfulness‐based interventions will bring significant added value to the existing mental health promotion programs and policy implications in the special schools.

The major study limitation of this study was the absence of a control group, which limited the ability to draw causal conclusions about the effects of the intervention. We considered including a control group; however, recruiting a control group is challenging due to the ethical concerns regarding withholding intervention from a control group in this vulnerable population. Additionally, it was not logistically feasible to perform randomization considering the lack of manpower to monitor or care for SEND children and adolescents in different groups and the inflexible schedules of schools for our activities.

In conclusions, the findings of this study contribute valuable insights to the field of special education on promoting mental health programs with mindfulness‐based interventions for SEND children and adolescents. The SWELE program's play‐based approach, combining unstructured outdoor activities with mindfulness‐based interventions, appears to be a beneficial strategy for promoting mental wellbeing and emotional competency in this population with special educational needs.

Future research could confirm the effectiveness and long‐term impacts of the mental health program and its potential for wider implementation across different educational settings and other population groups. Future research should focus on rigorous, large‐scale trials to confirm the SWELE program's effectiveness on promoting mental health, its long‐term impacts on students, and the best strategies for sustainable implementation across diverse educational settings and other populations. Research should also investigate how to adapt programs for different contexts, identify key implementation determinants like stakeholder engagement and school leadership, and develop strategies for continuous quality improvement and long‐term sustainability after initial implementation.

## Author Contributions

Regina LT Lee wrote the manuscript, Sally Wai Chi Chan, Yuen Yu Chong and Wai tong Chien reviewed the manuscript, Alice Wai Yi Leung and Kai Chow Choi conducted data analysis. All authors read and approved the final manuscript.

## Funding

The SWELE program is funded by Phase II of the Mental Health Initiatives Funding Scheme (MIH_0022), which is granted by the Food and Health Bureau, the Government of Hong Kong Special Administrative Region.

## Disclosure

Results (Abstract) have been submitted for review at the 7th Australian Nursing and Midwifery Conference 2025 for oral presentation.

## Ethics Statement

This study was reviewed and approved by the Joint Chinese University of Hong Kong and New Territories East Cluster Clinical Research Ethics Committee prior to conducting the study (CREC Ref. No: 2022.659‐T).

## Conflicts of Interest

The authors declare no conflicts of interest.

## Data Availability

Data is available upon reasonable request from the corresponding author and is otherwise restricted for ethical considerations, as it was instructed by the ethical committee.
